# Molecular dating of phylogenetic divergence between *Urochloa* species based on complete chloroplast genomes

**DOI:** 10.1186/s12864-017-3904-2

**Published:** 2017-07-06

**Authors:** Marco Pessoa-Filho, Alexandre Magalhães Martins, Márcio Elias Ferreira

**Affiliations:** 1 Embrapa Cerrados, BR 020, Km 18, Planaltina, Brasília, 73310-970 DF Brazil; 2 Laboratório de Genética Vegetal, Embrapa Recursos Genéticos e Biotecnologia, CP 02372 Brasília, Parque Estação Biológica, 70770-900 DF, Brazil; 3 Embrapa Labex USA, Agricultural Research Service, United States Department of Agriculture, Beltsville Agricultural Research Center, Bldg 006 Rm 200, 10300 Ave, Belstville, Baltimore, MD 20705 USA

**Keywords:** cpDNA, Brachiaria, Plastid, Phylogeny, Chloroplast markers

## Abstract

**Background:**

Forage species of *Urochloa* are planted in millions of hectares of tropical and subtropical pastures in South America. Most of the planted area is covered with four species (*U. ruziziensis, U. brizantha, U. decumbens* and *U. humidicola*). Breeding programs rely on interspecific hybridizations to increase genetic diversity and introgress traits of agronomic importance. Knowledge of phylogenetic relationships is important to optimize compatible hybridizations in *Urochloa*, where phylogeny has been subject of some controversy. We used next-generation sequencing to assemble the chloroplast genomes of four *Urochloa* species to investigate their phylogenetic relationships, compute their times of divergence and identify chloroplast DNA markers (microsatellites, SNPs and InDels).

**Results:**

Whole plastid genome sizes were 138,765 bp in *U. ruziziensis,* 138,945 bp in *U. decumbens*, 138,946 bp in *U. brizantha* and 138,976 bp in *U. humidicola*. Each *Urochloa* chloroplast genome contained 130 predicted coding regions and structural features that are typical of Panicoid grasses. *U. brizantha* and *U. decumbens* chloroplast sequences are highly similar and show reduced SNP, InDel and SSR polymorphism as compared to *U. ruziziensis* and *U. humidicola*. Most of the structural and sequence polymorphisms were located in intergenic regions, and reflected phylogenetic distances between species. Divergence of *U. humidicola* from a common ancestor with the three other *Urochloa* species was estimated at 9.46 mya. *U. ruziziensis*, *U. decumbens*, and *U. brizantha* formed a clade where the *U. ruziziensis* lineage would have diverged by 5.67 mya, followed by a recent divergence event between *U. decumbens* and *U. brizantha* around 1.6 mya.

**Conclusion:**

Low-coverage Illumina sequencing allowed the successful sequence analysis of plastid genomes in four species of *Urochloa* used as forages in the tropics. Pairwise sequence comparisons detected multiple microsatellite, SNP and InDel sites prone to be used as molecular markers in genetic analysis of *Urochloa.* Our results placed the origin of *U. humidicola* and *U. ruziziensis* divergence in the Miocene-Pliocene boundary, and the split between *U. brizantha* and *U. decumbens* in the Pleistocene.

**Electronic supplementary material:**

The online version of this article (doi:10.1186/s12864-017-3904-2) contains supplementary material, which is available to authorized users.

## Background

Forage grasses belonging to four species of *Urochloa* (previously included in *Brachiaria*) represent 85% of planted pastures in Brazil [1], extending over 115 Mha [2]. These pastures feed 90% of the commercial cattle herd raised in the country, which added up to 209 million heads in 2010 [3]. While *U. brizantha*, *U. decumbens*, and *U. humidicola* are the main species used as forages, interest in *U. ruziziensis* has grown due to its recent use in crop-livestock integrated systems, which could restore 18 Mha of degraded pastures in the next few years [2]. The four species are native to Africa and distributed in the humid and sub-humid tropics in South-East Asia, the Pacific Islands, Northern Australia and South America [4]. While *U. brizantha*, *U. decumbens*, and *U. humidicola* are predominantly polyploid and apomictic, *U. ruziziensis* is a sexual diploid species. The genus includes 110 species, and it is the largest of subtribe Melinidinae [5]. Inclusion of species in either *Brachiaria* or *Urochloa* has changed over time [6], and many research groups - forage breeders in particular - still refer to them as *Brachiaria*.

Phylogenetic relationships between the four main species of *Urochloa* have been subject of some controversy. Phylogenetic analysis of *Urochloa* species based on morphological traits included *U. ruziziensis*, *U. brizantha*, and *U. decumbens* in a group with *U. eminii*, *U. dura*, and *U. oligobrachiata* [7], a pattern that in part has not been confirmed in phylogenies based on molecular data. Analysis of ITS nuclear rDNA [6] clustered *U. brizantha* and *U. ruziziensis* in a clade with *U. comata* and *U. dura*, while *U. decumbens* was included in a group with *U. subulifolia*, *Melinis repens*, and *U. eruciformis*. *U. humidicola* joined another group, which included *U. dictyoneura* and *B. leersioides*. These relationships were maintained when molecular and morphological data were combined. Analysis of chloroplast DNA (cpDNA) regions*,* however, grouped *U. ruziziensis*, *U. brizantha*, and *U. decumbens* in a strongly supported clade [5], while *U. humidicola* was included in a separate clade with *U. dictyoneura* and *U. dura*.

Analyses of plastid genomes traditionally involve laborious isolation of chloroplasts, extraction and purification of plastid DNA, followed by sequencing and assembly [8–10]. New sequencing technologies have allowed investigation of plastid genomes in a more cost-effective, time-saving manner, with huge increases in sequence throughput [11–14]. This resulted in the publication of a growing number of plastid genomes of species for which genomic information was scarce or even absent. Examples in recent literature include species of bamboo [12], coconut palm [15] and the *Lolium-Festuca* complex [16]. Chloroplast genome sequencing and assembly also benefit research groups focused on chloroplast transformation for crop improvement [17–19]. Studies on structural and sequence variation in chloroplast genomes have contributed to plant phylogeny, ecology, comparative genomics, population genetics and evolution, particularly in angiosperms [20–22].

Chloroplast DNA ranges between 120 and 160 kb in size in most plants, with each chloroplast containing multiple copies of a circular chromosome composed of four regions: Large Single Copy (LSC), Small Single Copy (SSC), and two copies of an Inverted Repeat (IRa and IRb) [23]. Chromosomal organization, as well as the linear order of genes in cpDNA, vary little in angiosperms [23, 24]. Nucleotide substitution rates are low in coding regions of cpDNA, due to strong selection on the photosynthetic machinery, which restricts nucleotide mutation rates [24, 25]. It is possible, however, to detect structural and sequence variations that can be useful for phylogenetic analysis [26–28]. Use of cpDNA in phylogenetic analyses is also favored by the abundance of cpDNA after DNA extraction from leaf tissue [29, 30], by its usually maternal inheritance [31], and by the absence of recombination [32]. Structural and sequence variations in chloroplasts include single-nucleotide polymorphisms (SNPs), insertion-deletions (InDels), as well as microsatellites. Chloroplast microsatellites or SSRs (Simple Sequence Repeats) can be useful tools for the conservation and use of plant genetic resources. Chloroplast SSRs usually show polymorphism, and are generally composed of poly-A or poly-T sequences of approximately 20 bp [33–35]. Mononucleotide chloroplast SSRs have been used in studies of plant population structure and diversity, as well as in maternity tests [17, 34, 35]. Chloroplast SNPs and InDels have been recently applied to cultivar and food product differentiation in ginseng [36], germplasm identification in cacao [37], and species differentiation and identification in grasses [38, 39], and *Populus* [40].

Although sequence data generated by phylogenetic studies in *Urochloa-Brachiaria* is available on public databases, complete chloroplast genome sequences for members of these genera have not been published to date. Whole cpDNA sequence analysis could be used to investigate the phylogenetic relationships of the four cultivated species, identify chloroplast DNA markers (SSRs, SNPs and InDels), estimate their time of divergence and contribute to the current understanding of the *Urochloa* genus. This information is important for breeding programs which rely on interspecific hybridizations to increase genetic diversity and to introgress traits of agronomic importance in *Urochloa*. Therefore, the objectives of this study were to sequence, assemble, annotate, and compare complete chloroplast genomes of four species of *Urochloa* used as tropical grass forages. This data was then used to investigate phylogenetic relationships between these species based on complete cpDNA sequences, to estimate their times of divergence and to describe polymorphic cpDNA regions which will be useful for genetic analysis of *Urochloa*.

## Methods

### Plant material and DNA sequencing

Genomic DNA from samples of the four *Urochloa* species was extracted using a standard CTAB protocol [41] with modifications [42]. Samples included a selfed clone of *U. ruziziensis* cv. Kennedy (FSS-1) [43]; *U. brizantha* BRS cv. Marandu; *U. decumbens* cv. Basilisk; and *U. humidicola* cv. Tupi. Genomic libraries were prepared according to manufacturer’s instructions (Illumina, San Diego, CA, USA). In summary, DNA fragments were obtained by nebulization, and their 3′ ends were added an adenine, to which adapter fragments were ligated. Ligation products were run on an 1% agarose gel, and fragments of ~200 bp were excised and purified. Sequencing of each paired-end genomic DNA fragment library was performed on the Illumina GAII sequencer, with six sequencing lanes for *U. ruziziensis* and one lane each for the other three species.

### Chloroplast genome assembly

FASTQ formatted files containing DNA sequencing reads were submitted to the short-read correction tool of SOAP*denovo* (Release 1.05), designed to correct Illumina GA reads [14]. The KmerFreq and ErrorCorrection routines were run with default parameters (seed length = 17, quality cutoff = 5). Illumina sequencing adapters and low quality reads were eliminated using the CLC trimmer function (default limit = 0.05) (CLC Genomics Workbench 4.1 software, CLC Bio, Aarhus, Denmark). Error corrected FASTQ files were then submitted to assembly routines performed on CLC Genomics. Reads from the four *Urochloa* species were initially assembled using the *Panicum virgatum* cv. Summer chloroplast genome (NC_015990) as a reference, with assembly routines of the CLC Genomics Workbench. High quality and matching reads (e-value = −10) were initially selected for assembly. Additionally, four de novo assemblies of the cpDNA molecules of each *Urochloa* species were also performed and compared to results of assemblies using *P. virgatum* as reference. In this case, sequence reads BLASTed against *P. virgatum* were submitted to assembly routines performed on CLC Genomics with de novo assembly using paired-end reads. Bubble size was automatically defined by the software as 50 bp. Assembly Length Fraction and Similarity parameters were set to 0.5 and 0.8, respectively. Mismatch, deletion and insertion cost parameters were set to 2, 3 and 3, respectively. The k-mer size on CLC Bio assembler was set to 25 bp and the coverage cutoff to 1000X.

### Chloroplast genome sequence analysis

Annotation was performed using DOGMA [44] with default parameters. Predicted coding regions were manually adjusted for their start and stop codons, after inspection and comparison with available chloroplast genomes in tribe Paniceae. Corrections were made using Sequin (http://www.ncbi.nlm.nih.gov/Sequin/) and Artemis [45]. Graphic representations of the annotated plastid genomes were obtained with OGDraw [46]. Complete chloroplast genomes from nine species in tribe Paniceae were compared regarding their levels of sequence conservation, using the Multi-LAGAN alignment program [47] included in mVISTA [48, 49], with default parameters. The chloroplast genome sequence of *U. humidicola* was used as a reference for these alignments. In addition to the sequences of four *Urochloa* species from this study, complete plastid sequences from switchgrass (*Panicum virgatum*, NC_015990), pearl millet (*Cenchrus americanus*, NC_024171), foxtail millet (*Setaria italica*, NC_022850), late barnyard grass (*Echinochloa oryzicola*, NC_024643), and white fonio (*Digitaria exilis*, NC_024176) were included in this analysis.

Assembled chloroplast sequences were analyzed with the perl script MISA [50] for the detection of microsatellite regions. Parameters included searches for regions with repeat units ranging from one to six. Thresholds for a minimum number of repeat units were established as follows: at least 10 repeat units for mononucleotide regions; five repeat units for dinucleotides; three repeat units for tri- and tetranucletides; and five repeat units for penta- and hexanucleotides.

SNPs and InDels were detected from pairwise alignments of complete plastid sequences using the NUCmer program included in MUMmer v 3.23 [51]. A perl script was used to parse output files from NUCmer and produce VCF files containing SNP and InDel information from each pairwise comparison (mummer2Vcf.pl script available at https://github.com/marcopessoa/bioinfo-scripts). The programs snpEff and SnpSift v. 4.2 [52, 53] were used to annotate genomic regions and types of effects of the detected SNPs and InDels. For each pairwise comparison, effects were annotated using the most ancestral species as a reference, based on results of phylogenetic analyses.

### Phylogenetic analyses

Plastid nucleotide sequences from the four *Urochloa* species and from 26 other Poaceae species were aligned using parallel MAFFT v. 7.187 [54] on XSEDE [55] via the CIPRES Science Gateway [56]. Alignments included the LSC region, only one copy of the IR, and the SSC region of each plastid. The FFT-NS-i [57] executable was used, with 1000 cycles of iterative refinement. The list of species included in this analysis is presented on Additional file 1. This dataset was analyzed using Maximum Parsimony (MP), Maximum Likelihood (ML, [58]), and Bayesian Markov Chain Monte Carlo inference (BI, [59]).

MP analyses were performed with PAUP*4.0a144 [60], using an initial Heuristic Search with 1000 random-taxon-addition replicates, the tree-bisection-reconnection (TBR) branch-swapping algorithm, and the “MulTrees” option in effect. Non-parametric bootstrap [61] was applied with 10,000 replicates, each with 10 random-taxon-addition replicates. ML analyses was performed on RAxML-HPC2 on XSEDE [62] using the GTRGAMMA model for the rapid bootstrapping phase, and *Puelia olyriformis* as an outgroup. The search for the best-scoring ML tree and the rapid bootstrap were performed in a single run. Bootstrapping was stopped automatically with the autoMRE Majority Rule Criterion, and was followed by ML optimization steps. BI was conducted using MrBayes v. 3.2.3 [63], with two independent runs and four chains. Each run was performed until completion, and included 20,000,000 generations, with sampling every 100 generations. The first 3000 generations were discarded as burn-in. Trees were visualized with FigTree v1.4.2 [64].

### Divergence time estimation

The ML tree obtained with RAxML was used as input to generate an ultrametric tree with the *chronos* function of the R package *ape* [65], (lambda = 0, a relaxed model, and a calibration for the most recent common ancestor [MRCA] of rice and maize with a minimum age of 32 and a maximum age of 66 million years). These constraints were based on phytolith data from the North American Great Plains [66], which suggest that BEP and PACMAD clades had diverged by 35 mya [67]. The resulting ultrametric tree was used as the starting tree for BEAST v2.3.1 [68] runs, which was used for molecular dating. BEAST parameters were set under an uncorrelated log-normal clock model [69], a Calibrated Yule tree prior, and the GTR substitution model. Site model parameters included four gamma categories, a value of 1.0 for the gamma shape distribution, a proportion of invariant sites set to 0.5, and an empirical frequency model. A prior for the calibration of the MRCA node for the BEP-PACMAD group was included following an exponential distribution with mean 20.0 and offset 35.0. The BEP-PACMAD group was constrained as monophyletic during MCMC analysis. Each MCMC run had a chain length of 100,000,000 iterations, with sampling every 10,000 steps. An input file with all analyses parameters was prepared using BEAUTi v2.3.1, and is included as Additional file 2. Convergence between two MCMC runs, as well as their respective means and ESS values for logged statistics were assessed using Tracer v1.6.0 [70]. Tree and log files were combined with Logcombiner v2.3.1.

## Results and discussion

### Chloroplast genome sequencing, assembly, and annotation

Input data from *U. ruziziensis* was 3X greater than that from the other species, but coverage and proportion of mapped reads on the reference *P. virgatum* chloroplast genome did not increase proportionally, since the cpDNA molecule is small. In *U. brizantha*, for instance, a higher proportion (4%) of reads were mapped on the *P. virgatum* chloroplast genome sequence, while for the other species this value ranged between 1 and 2% of all sequence reads (Table 1). This resulted in an average coverage in *U. brizantha* (2791X) that was higher than that observed for *U. ruziziensis* (2011X). The high average coverage values for the assembled contigs, all of which were greater than 1000X (Table 1), did not seem to be related to the initial number of reads obtained for each species, given that *U. brizantha* had a higher mean coverage. It seems, though, that *U. brizantha* whole DNA extraction had proportionally more cpDNA sequences than the other species. A possible explanation for this observation should still be pursued.

**Table 1:** Assembly metrics of four chloroplast genomes of *Urochloa* forage species, using the *P. virgatum* plastid sequence as a reference

Species	Total reads (bp)	Reads mapped to *P. virgatum* cpDNA (bp)	% of mapped reads	cpDNA assembly size (bp)	LSC size (bp)	SSC size (bp)	IRa size (bp)	IRb size (bp)	Mean coverage
*U. ruziziensis*	20,211,010,448	279,025,488	1%	138,765	80,798	12,537	2715	22,715	2011
*U. brizantha*	8,643,705,720	387,850,876	4%	138,946	81,008	12,535	22,699	22,704	2791
*U. decumbens*	9,018,811,776	168,717,644	2%	138,945	81,005	12,537	22,699	22,704	1321
*U. humidicola*	8,476,910,040	183,602,548	2%	138,976	81,017	12,535	22,711	22,713	1214
*P. virgatum*	-	-	-	139,619					

Annotated chloroplast genome sequences were submitted to GenBank and are available under accession numbers NC_030066-NC030069. The four chloroplast genomes showed a typical circular chromosome including the LSC region (ranging from 80,798 bp in *U. ruzizienis* to 81,017 bp in *U. humidicola*), the SSC region (12,535 in *U. brizantha* and *U. humidicola*; 12,537 bp in *U. ruziziensis* and *U. decumbens*), and the two IR regions (ranging from 22,699 in *U. brizantha* and *U. decumbens* to 22,715 bp in *U. ruziziensis*) (Table 1). The two IR regions had the same size in *U. ruziziensis*, but differed by 2 bp in *U. humidicola*, and by 5 bp in *U. brizantha* and *U. decumbens*. Whole plastid genome sizes ranged between 138,765 bp in *U. ruziziensis* to 138,976 bp in *U. humidicola*, all genomes sequenced being smaller than the reference chloroplast genome (139,619 bp in *Panicum virgatum*) (Table 1). Size differences between assemblies for *U. brizantha* and *U. decumbens* reached only 1 bp, going up to 211 bp between *U. humidicola* and *U. ruziziensis*. De novo assemblies of each cpDNA molecule represented 92.89 to 99.45% of chloroplast genomes assembled using *P. virgatum* cpDNA as reference (Additional file 3). Sequence alignment corroborated the results obtained using *P. virgatum* as reference.

**Fig. 1:**
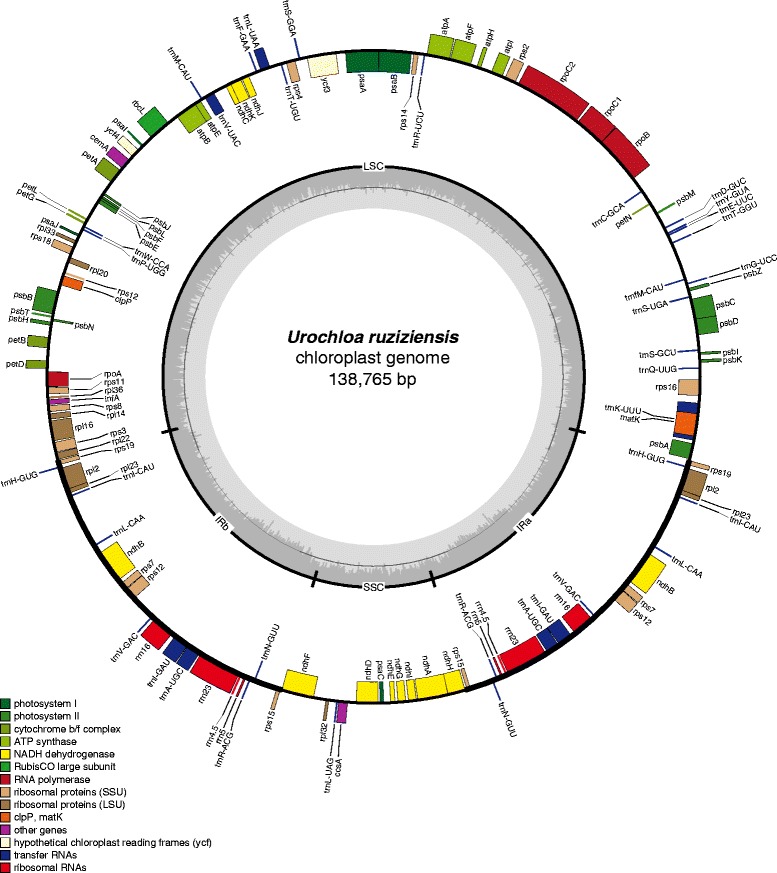
Genome structure and mapping of genes in the *U. ruziziensis* chloroplast genome. The LSC, SSC, and IR regions are labeled accordingly. The inner circle depicts GC content. Genes on the outside of the map are in the forward strand, while genes on the inside are in the reverse strand. Genes are colored according to their functions as shown in the legend

Each of the four *Urochloa* chloroplast genomes contained 130 predicted coding regions, 112 of which were unique, and 18 of which were duplicated in the two IRs. These regions included 77 protein-coding genes, 31 tRNAs, and 4 rRNAs. Eleven protein-coding genes and seven tRNAs contained introns. Coding regions ranged between 51.6% and 51.8%, and GC contents ranged between 38.5 and 38.63%. Figure 1 shows the genome structure and mapping of these genes on the *U. ruziziensis* chloroplast genome. Typical features of chloroplast genome organization of Panicoid grasses were found, such as the loss of genes *accD*, *ycf1*, and *ycf2* [71]. The IR regions also contained *trnH-GUG* and *rps19* near the IR-LSC junction [71, 72]. The IRb/SSC boundary included 29-bp of the *ndhF* gene in IRb, a feature that is unique to subfamily Panicoideae [71]. Recent reports have shown the feasibility of fully assembling chloroplast genomes de novo using either Illumina short reads [73] or PacBio single-molecule real-time (SMRT) sequences. This will certainly increase the number of large scale phylogenomic studies using either complete chloroplast sequences [74] or single nucleotide variants (SNVs) [75].

**Fig. 2:**
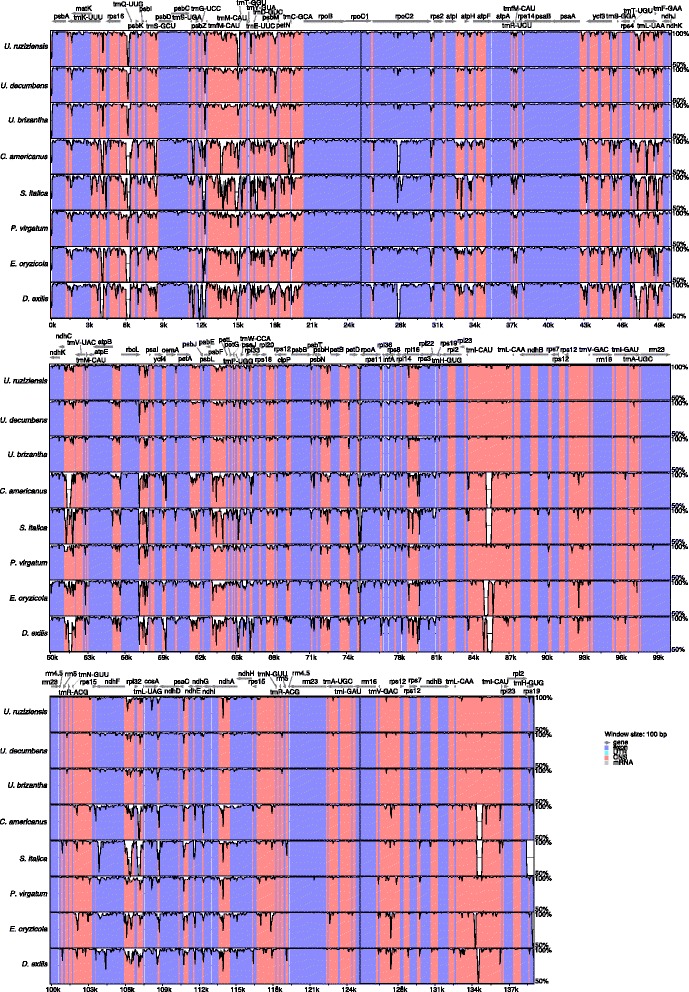
Alignment and percentage identity of complete chloroplast sequences of *Urochloa* and five species of Paniceae using mVISTA. Measures of similarity are relative to *U. humidicola*, which was used as a reference. Coding regions are shaded in blue, while noncoding sequences are shaded in pink. *Arrows* indicate positions of annotated genes in *U. humidicola*

Comparative analysis between the four *Urochloa* chloroplast genomes showed high values of sequence conservation between *U. ruziziensis*, *U. brizantha* and *U. decumbens* when compared to *U. humidicola* (Fig. 2). Values are close to 100% in coding regions, with very few regions of lower similarity (between 50% and 60%) in non-coding regions. On average, *U. humidicola* had 98.3% sequence similarity with *U. decumbens* and *U. brizantha*, and 98.2% with *U. ruziziensis*. As expected, sequence conservation decreases when *U. humidicola* is compared to more distantly related species in tribe Paniceae (Fig. 2). However, average similarity still ranged between 94.9% between *U. humidicola* and *Digitaria exilis* and 97.2% between *U. humidicola* and *P. virgatum*.

### Chloroplast molecular markers

#### Microsatellites

The number of cpSSRs detected in the four *Urochloa* species ranged between 80 in *U. decumbens* and 84 in *U. brizantha* (Table 2). Loci with tri-nucleotide repeat motifs were the most abundant, followed by those with mono-nucleotide repeats. *U. ruziziensis* presented the largest mono-nucleotide locus, with 24 bp, located at position 44,778 bp of intron 1 of *ycf3*. The number of mono-nucleotide repeat loci ranged between 24 in *U. decumbens* and *U. humidicola* and 29 loci in *U. ruziziensis*. These values are in agreement with what was described for *P. virgatum* [17], with 25 mono-nucleotide microsatellite loci of length 10 bp or greater in that species. In common wheat, 24 loci having more than ten mononucleotide repeats have been detected [76].

**Table 2:** Number of microsatellites with mono-, di-, tri-, and tetra-nucleotide repeat motifs in chloroplast genomes of four *Urochloa* species

Unit size	Species			
	*U. brizantha*	*U. decumbens*	*U. ruziziensis*	*U. humidicola*
1	28	24	29	24
2	4	5	5	5
3	42	41	39	42
4	10	10	9	10
Total	84	80	82	81
Largest mononucleotide repeat (bp) / Location (bp)	15 / 50,621	21 / 30,776	24 / 44,778	17 / 45,087

We looked for inter-specific cpSSR polymorphisms between the four *Urochloa* chloroplast genomes using in silico analysis (Additional file 4). Out of 84 cpSSRs detected in *U. brizantha*, for instance, 32 sites were potentially polymorphic in at least one of the other three *Urochloa* species. Forty-five cpSSRs were located in genic regions, 38 of which were in exons (Additional file 4). The gene with the largest number of cpSSRs was *rpoC2*, with seven loci. One trinucleotide locus detected in *U. brizantha* and *U. decumbens* (trnfM-CAU-trnT-GGU intergenic region, position 15,138 bp in *U. brizantha*) was absent in *U. ruziziensis* and *U. humidicola* (Additional file 4). Three other loci presented changes in their repeat motifs, which modified their status from perfect to imperfect cpSSRs. The usefulness of these loci for intra- and inter-specific analyses remains to be experimentally validated in a future study. Potential applications include studies of intra-specific plant population structure and diversity, as well as maternity tests which could be useful in breeding programs based on the generation of intra- and inter-specific hybrids. The first set of nuclear microsatellite markers for *U. ruziziensis,* using a draft assembly of its nuclear genome from Illumina sequence data, has been recently published and applied to genetic analysis of *U. ruziziensis* [43, 77].

#### Single nucleotide polymorphisms (SNPs) and insertion/deletion (InDel) sites

Pairwise sequence comparisons allowed the identification of SNPs in the *Urochloa* chloroplast genomes, with numbers ranging from 170 SNPs between *U. brizantha* and *U. decumbens*, up to 1338 SNPs between *U. decumbens* and *U. humidicola* (Table 3). Most of the detected SNPs in all pairwise comparisons were located in intergenic regions. The number of chloroplast SNPs detected between *U. brizantha* and *U. decumbens* is 4.5× smaller than between *U. brizantha* and *U. ruziziensis*, and almost 8× smaller than between *U. brizantha* x *U. humidicola*. A similar numbers were observed in comparisons between *U. decumbens* and *U. ruziziensis*, as well as between *U. decumbens* and *U. humidicola* (Table 3). Approximately 28% of the SNP effects, i.e., mutations with potential effect on gene expression, are located in genic regions in the *U. brizantha* x *U. decumbens* comparison, while this number increases up to 41% in other comparisons. Thirty-nine SNPs located in exons were detected between *U. brizantha* x *U. decumbens* (19 of which were missense mutations using *U. brizantha* as a reference). This number increases in other pairwise comparisons and it was found to be as high as 439 SNPs between *U. decumbens* x *U. humidicola* (144 of which were missense mutations using *U. humidicola* as reference). Similar pattern was observed in tRNA loci mutations, which were about 2% of the total number of SNP effects detected between *U. brizantha* x *U. decumbens*, and up to 7.1% between *U. ruziziensis* x *U. humidicola*. Additional file 5 includes SNPs detected in genes for all pairwise comparisons and their respective positions in base-pairs. The three genes with the largest numbers of SNPs in exons were *rpoC2*, *ndhF*, and *matK*, for all pairwise comparisons but one: *rpoC2* was followed by *ccsA* and *rps18* when *U. brizantha* and *U. decumbens* were compared.

**Table 3:** Total number of SNPs, their locations (inter or intragenic), and effects in pairwise comparisons of four *Urochloa* chloroplast genomes

Pairwise comparison	*U. brizantha* *x* *U. decumbens*	*U. ruziziensis* *x* *U. decumbens*	*U. ruziziensis* *x* *U. brizantha*	*U. humidicola* *x* *U. ruziziensis*	*U. humidicola* *x* *U. brizantha*	*U. humidicola* *x* *U. decumbens*
Total number of SNPs	170	752	788	1319	1307	1338
Number of Effects	172	767	805	1356	1343	1371
Intergenic	119	418	440	734	749	772
Intragenic	49	315	328	526	502	511
Intron	10	52	61	82	66	72
Exon	39	263	267	444	436	439
synonymous coding mutation	20	174	175	305	293	296
non-synonymous coding mutation	19	89	92	140	144	144
Missense	19	89	92	139	144	144
Nonsense	-	-	-	1	-	-
Other (tRNAs)	4	34	37	96	92	88

Pairwise cpDNA sequence comparisons also allowed the identification of InDels between the four *Urochloa* species. The lowest number of InDels (91) was found between *U. brizantha* and *U. decumbens*, while the largest number (259) was found between *U. brizantha* and *U. humidicola*. Results for all pairwise comparisons are shown in Additional file 6. A high correlation between the number of identified InDels and SNPs between species was detected (0.996). The number of InDels located in chloroplast genic regions ranged from 12 between *U. decumbens* and *U. brizantha,* to 30 between *U. humidicola* and *U. ruziziensis*. When all pairwise comparisons are considered, these InDels were mapped on genes *rpoC2*, *ccsA*, *rbcL*, *rps18*, and *ndhK*.

The chloroplast gene *rpoC2* presented the largest numbers of cpSSRs, SNPs and InDels. This gene codes for the β” subunit of RNA polymerase, and is well known as a hotspot of structural and sequence variation in chloroplasts of grass species [78]. Recently, PCR markers based on *rpoC2* structural variation were developed for differentiation and identification of species used in commercial food products [38]. InDels detected in *Urochloa* chloroplasts could be easily deployed as markers for species differentiation and identification. These would be useful, for instance, for identification of accessions in germplasm collections, and confirmation of hybridizations in breeding programs.

### Phylogenetic analyses

**Fig. 3:**
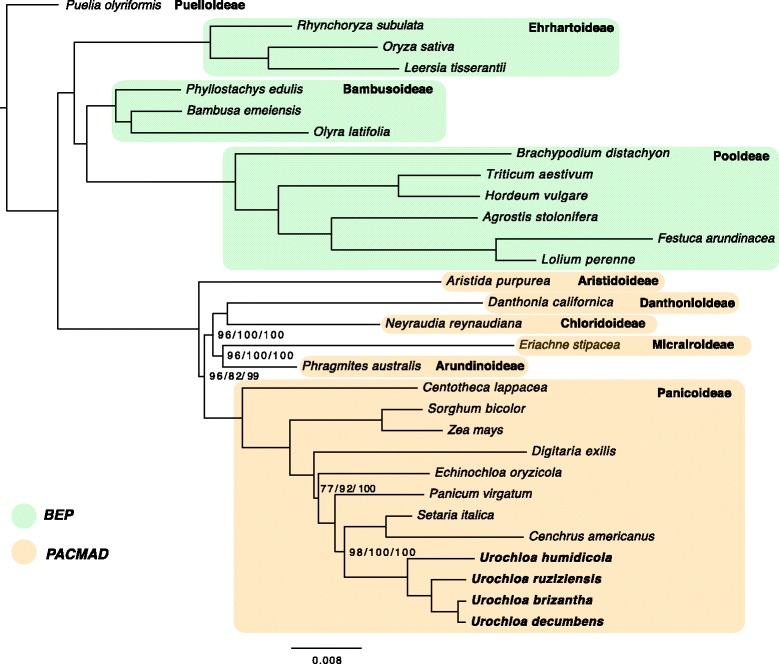
Maximum-likelihood tree based on complete chloroplast sequences of 30 species of Poaceae. Unlabeled nodes had supporting values of 100%. Where noted, supporting values are shown in the following order: MP/ML/BI

Phylogenetic analysis using plastid nucleotide sequences of 30 species of Poaceae resulted in trees with well supported clades. The MP tree was built using 16,322 parsimony-informative characters, and the score of the best MP tree was 57,653. The best scoring ML tree had a Likelihood of - 526,037.12. The BI analysis showed that runs reached stationarity, and convergence diagnostics showed that parameters were properly sampled, with most of the Estimated Sample Size (ESS) values above 200. Tree topologies for MP, ML, and BI analyses were identical, and results are presented and discussed using the best scoring ML tree (Fig. 3) including support values for each method when they were below 100%.

Using *Puelia olyriformis* as an outgroup, the split between the BEP and PACMAD clades was clearly observed with 100% support. *Puelia* is one of two genera belonging to the Puelioideae, a deeply diverging grass subfamily endemic to tropical forests of West Africa [79]. Nodes were strongly supported with bootstrap values above 90%, with the following exceptions: (i) the MP tree showed a bootstrap value of 72% for divergence of *Echinochloa oryzicola* from *Panicum*, *Setaria*, *Cenchrus* and *Urochloa*; (ii) support for the relationship of Panicoideae to other members of the PACMAD clade was 82% in the ML tree. Relationships in the PACMAD clade were in agreement with those found by the Grass Phylogeny Working Group II [80]. In summary, Aristidoideae species were shown as the first diverging clade in PACMAD, sister to other subfamilies, and followed by Panicoideae. In a recent study using fully assembled plastome sequences [73], Panicoideae species were grouped as a sister clade to all other PACMAD grasses (with Aristidoideae being sister to the CMAD clade), but results were not statistically different from what was described by GPWG II. Taxon sampling in our study is small and may lead to artifactual groups [73]. However, increase in character sampling from the use of full chloroplast genome sequences results in strong support of phylogenetic relationships [73]. This is evident from the agreement with topologies of larger studies such as the one performed by GPWG II, in addition to strong support values and consistency between different phylogenetic analysis methods.

Inside Paniceae, *Digitaria exilis* (subtribe Anthephorinae) was the earliest diverging species, followed by *Echinochloa oryzicola* (subtribe Boivinellinae), and *Panicum virgatum* (subtribe Panicinae). Finally, *Setaria italica* and *Cenchrus americanus* (both members of subtribe Cenchrinae) appeared in a sister group to the four *Urochloa* species (subtribe Melinidinae). These results are also in agreement with those found by recent phylogenies [67, 80, 81], and confirm that the use of full plastome sequences can lead to well supported and consistent phylogenetic reconstructions.

Grouping of the four *Urochloa* species was consistent with what was found in a previous study [5] using *rpl16*/*trnL* intron/*trnL-F* spacer/*ndhF* sequences: *U. ruziziensis*, *U. brizantha*, and *U. decumbens* were grouped in a strongly supported clade, while *U. humidicola* was shown as a sister taxon. In another study using *rpl16* and *ndhF*, *U. brizantha* and *U. decumbens* were also found to be closely related, and separated from *U. humidicola* and *U. dictyoneura* [82]. Interestingly, the first published molecular phylogeny for *Urochloa* and *Brachiaria*, using ITS nuclear ribosomal DNA analysis [6], showed different results: *U. brizantha* and *U. ruziziensis* were included in a clade with *U. comata* and *U. dura*, while *U. decumbens* was included in a group with *U. subulifolia*, *Melinis repens*, and *U. eruciformis*. These relationships were maintained when molecular and morphological data were combined [6]. Contrasting these findings, morphological analysis alone had included *U. ruziziensis*, *U. brizantha*, and *U. decumbens* in a group with *U. eminii*, *U. dura*, and *U. oligobrachiata* [7].

The grouping of *U. ruziziensis*, *U. decumbens*, and *U. brizantha* is also consistent with their belonging to an agamic complex-a group of species considered as being distinct, and reproductively isolated by ploidy levels and apomixis [7, 83, 84] (see next section). This reproductive barrier, however, can be overcome by polyploidization of sexual diploids, allowing inter-specific hybrid production, a strategy that is currently applied in *Urochloa* breeding programs in Brazil and at the International Center for Tropical Agriculture (CIAT, Colombia) [7, 85, 86]. Results presented in the previous section regarding the presence of SNPs and InDels between these species pointed in the same direction. The number of inter-specific structural and sequence polymorphisms also indicated that *U. ruziziensis* is more closely related to *U. brizantha* than to *U. humidicola*, and that chloroplast sequence similarity between *U. brizantha* and *U. decumbens* is high, given the lower number of chloroplast SNPs and InDels found between these two species.

### Divergence estimates

**Fig. 4:**
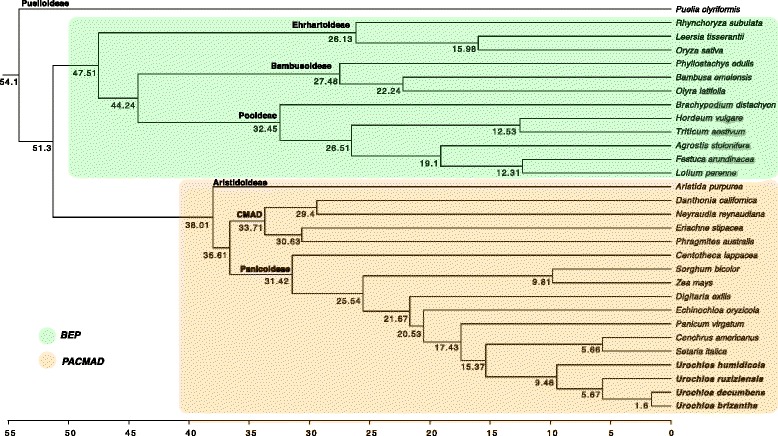
Divergence times of 30 species of Poaceae based on complete chloroplast sequences. Values at nodes indicate divergence dates in millions of years. The four *Urochloa* species are shown in bold

Divergence time estimates were based on a single calibration point at the BEP-PACMAD node using phytolith data, which suggests that all major grass subfamilies had diverged by 35 mya [66]. Results of divergence dates for some of the observed clades as well as the upper and lower bounds of the 95% highest posterior density (HPD) intervals are shown on Table 4. A complete chronogram is shown in Fig. 4. The estimated divergence date for Puelioideae was 54.1 [35.22, 92.11] mya, and the BEP-PACMAD divergence date was 51.3 [35, 86.8] mya. These results are in agreement with those found using the same phytolith calibration for BEP-PACMAD, and a combination of plastid *ndhF* and nuclear *phyB* sequence alignments [67]. Their divergence estimates for Puelioideae and for the BEP-PACMAD divergence were 52.6 mya, and 49 mya, respectively. Another recent study estimated the age of BEP-PACMAD to be 54.9 mya [87]. The estimated time of divergence for the crown PACMAD node was 38.1 [21.65, 65.71] mya, and is also in agreement with recent studies [67, 73, 87].

**Table 4:** Times of divergence of Poaceae clades based on BEAST analysis of complete chloroplast sequences, with a calibration for BEP-PACMAD divergence (exponential distribution, mean 20.0, offset 35.0)

Clade	Age (mya)	95% HPD Lower Bound	95% HPD Upper Bound
Puelioideae	54.1	35.22	92.11
BEP-PACMAD divergence	51.3	35	86.8
Aristida - Crown PACMAD	38.01	21.65	65.71
CMAD	33.71	18.36	58.48
Panicoideae	31.42	17.26	54.8
*Urochloa – Setaria*	15.37	6.66	27.82
*Urochloa humidicola*	9.46	3.94	17.35
*Urochloa ruziziensis*	5.67	2.04	10.79
*U. decumbens – U. brizantha*	1.6	0.36	3.43

With our taxon sampling, the date of the *Setaria-Urochloa* divergence was estimated at 15.37 [6.66, 27.82] mya. Divergence of *U. humidicola* from a common ancestor with the three other *Urochloa* species was estimated at 9.46 [3.94, 17.35] mya. In the clade composed by *U. ruziziensis*, *U. decumbens*, and *U. brizantha*, *U. ruziziensis* would have diverged by 5.67 [2.04, 10.79] mya, followed by a recent divergence event between *U. brizantha* and *U. decumbens* around 1.6 [0.36, 3.43] mya. The *Urochloa* clade had an estimated date of origin at 7.2 ± 2.2 mya in a previous study [67], which overlaps with the results found in our analysis. Our results place the origins of *U. humidicola* and *U. ruziziensis* separation in the Miocene-Pliocene boundary, and the split between *U. brizantha* and *U. decumbens* in the Pleistocene.

*U. ruziziensis*, *U. decumbens* and *U. brizantha* have traditionally been considered members of a single agamic complex [7, 83, 84] - a group of species that includes sexual diploids and polyploids among facultative or obligate apomicts, originated from hybridizations among sexual diploid and polyploid members [88]. These hybridizations would initially take place among sexual diploids, generating hybrids at different ploidy levels [88]. Gene exchange would still be possible for sexual triploids and tetraploids, but asexual reproduction in higher ploidy levels would lead to reproductive isolation and the occurrence of microspecies with discontinuous morphological variation. Indeed, while most accessions of *U. brizantha* and *U. decumbens* are tetraploid and predominantly apomict, a few diploid sexual accessions of *U. decumbens* can be found in germplasm collections [89–91] and at least one sexual diploid accession of *U. brizantha* is available [92, 93]. One hexaploid sexual accession of *U. humidicola* has also been reported [94].

The similarity between chloroplast genomes of *U. brizantha* and *U. decumbens* is striking. Their cpDNA sizes differ by just 1 bp, and the cpDNA polymorphism (SNPs, InDels, SSRs) detected on coding and intergenic regions is smaller than in pairwise comparisons with *U. ruziziensis* and *U. humidicola*. The average divergence time between *U. brizantha* and *U. decumbens* is estimated to be recent (1.6 mya). Given the high sequence similarity between their cpDNA genomes, these combined data would indicate that a single polyploidization event took place to establish the *U. brizantha* and *U. decumbens* lineages. However, complementary analysis of cpDNA sequence data of germplasm accessions of both species would be necessary to confirm this hypothesis.

The taxonomic complexity of the *Urochloa* genus is also characteristic of forage grass species in general [95]. Hybridization and allopolyploidization are probably common processes in *Urochloa,* leading to reticulate evolution events and to potential incongruences between nuclear and chloroplast phylogenies [5, 96, 97]. A recent paper on the phylogeny of photosynthesis in Paniceae using a combination of chloroplast, mitochondrial and nuclear rDNA found that phylogenies from different types of markers did differ in certain areas of the trees [81]. In order to further investigate taxonomic relationships between the species described here, the inclusion of accessions of *U. brizantha* and *U. decumbens* with different ploidy levels, especially the diploids, would be important. In addition to larger taxon sampling, a robust nuclear phylogeny [5] would be necessary to properly identify the most likely parent species of *Urochloa* polyploids used as forages.

## Conclusions

Use of low-coverage Illumina sequencing allowed the successful assembly and annotation of plastid genomes in four species of *Urochloa* extensively used as forages in the tropics (*U. ruziziensis*, *U. brizantha, U. decumbens* and *U. humidicola*). Comparative analyses of these chloroplast genomes allowed the identification of sequence and structural polymorphisms that will be useful for future genetic studies in *Urochloa* species. Results were consistent with previous phylogenies that group *U. ruziziensis*, *U. brizantha* and *U. decumbens* in a well-supported clade. *U. brizantha* and *U. decumbens* chloroplast sequences are highly similar and show reduced SNP, InDel and SSR polymorphism as compared to *U. ruziziensis* and *U. humidicola*. Future phylogenetic studies based on complete plastid sequences should include diploid samples of *U. decumbens* and *U. brizantha*, in addition to nuclear markers that could provide a better understanding of relationships between these species. The increased throughput and reduced costs of next-generation sequencing technologies bring the opportunity for the execution of phylogenetic studies based on either complete or large fragments of plastids, including a high number of taxa.

## Additional files

Additional file 1: List of Poaceae species with available plastid sequences used in phylogenetic analyses in this study, their respective tribes, and NCBI accession codes. (PDF 72 kb)

Additional file 2: XML input file used for analysis in BEAST. (TXT 3981 kb)

Additional file 3: Sequencing and de novo assembly metrics of chloroplast genomes of four *Urochloa* species. (PDF 13 kb)

Additional file 4: Annotation of chloroplast microsatellites in four *Urochloa* species. Genome locations are based on the plastid genome of *U. brizantha*. The table includes locations of cpSSRs relative to annotated genes, types of regions (exon, intron, intergenic), repeat motifs, physical position in bp, polymorphism information, and number of repeats in *U. decumbens*, *U. ruziziensis*, and *U. humidicola*. (PDF 44 kb)

Additional file 5: Annotation of SNPs located in genes in six pairwise comparisons between *Urochloa* species. The table includes genes, mutations, and SNP positions in bp. (PDF 46 kb)

Additional file 6: Total number of InDels, their effects, and genome locations in six pairwise comparisons between *Urochloa* species. InDels located in genes are listed with their respective variations (insertion/deletion) and genome positions. (PDF 23 kb)

## Abbreviations

BI: Bayesian inference
CIAT: Centro Internacional de Agricultura Tropical (International Center of Tropical Agriculture)
cpDNA: chloroplast DNA
cpSSR: chloroplast simple sequence repeat
CTAB: cetyl trimethylammonium bromide
ESS: estimated sample size
GPWG: Grass phylogeny working group
InDel: Insertion-Deletion
IRa: Inverted repeat A
IRb: Inverted repeat B
ITS: internal transcribed spacer
LSC: large single copy
MCMC: Markov chain Monte Carlo
Mha: million hectares
ML: maximum likelihood
MP: maximum parsimony
MRCA: most recent common ancestor
Mya: million years ago
PCR: polymerase chain reaction
rDNA: ribosomal DNA
SMRT: single-molecule real-time
SNP: single-nucleotide polymorphism
SNV: single nucleotide variant
SSC: small single copy
SSR: simple sequence repeat
TBR: tree-bisection-reconnection
