# Supplementary material for: Molecular dating of phylogenetic divergence between Urochloa species based on complete chloroplast genomes
Source: BMC Genomics. 2017 Jul 6;18:516. doi: 10.1186/s12864-017-3904-2 (PMC5499013; doi:10.1186/s12864-017-3904-2)
Supplement: Supplementary file 1 — List of Poaceae species with available plastid sequences used in phylogenetic analyses in this study, their respective tribes, and NCBI accession codes. (PDF 72 kb) [file 12864_2017_3904_MOESM1_ESM.pdf]

Additional file 1 – List of Poaceae species with available plastid sequences used in phylogenetic analyses in this study, their respective tribes, and NCBI accession codes.

**Species**	**Tribe**	**NCBI accession code**
Puelia olyriformis	Puelioideae	NC_023449
Rhynchoryza subulata	Ehrhartoideae	NC_016718
Leersia tisserantii	Ehrhartoideae	NC_016677
Oryza sativa	Ehrhartoideae	NC_001320
Phyllostachys edulis	Bambusoideae	NC_015817
Bambusa emelensis	Bambusoideae	NC_015830
Olyra latifolia	Bambusoideae	NC_024165
Brachypodium distachyon	Pooideae	NC_011032
Hordeum vulgare	Pooideae	NC_008590
Triticum aestivum	Pooideae	NC_002762
Agrostis stolonifera	Pooideae	NC_008591
Festuca arundinacea	Pooideae	NC_011713
Lolium perenne	Pooideae	NC_009950
Aristida purpurea	Aristidoideae	NC_025228
Danthonia californica	Danthonioideae	NC_025232
Neyraudia reynaudiana	Chloridoideae	NC_024262
Eriachne stipacea	Micraidoideae	NC_025234
Phragmites australis	Arundinoideae	NC_022958
Centotheca lappacea	Panicoideae	NC_025229
Sorghum bicolor	Panicoideae	NC_008602
Zea mays	Panicoideae	NC_001666
Digitaria exilis	Panicoideae	NC_024176
Echinochloa oryzicola	Panicoideae	NC_024643
Panicum virgatum	Panicoideae	NC_015990
Cenchrus americanus	Panicoideae	NC_024171
Setaria italica	Panicoideae	NC_022850
Urochloa humidicola	Panicoideae	NC_030069
Urochloa ruziziensis	Panicoideae	NC_030068
Urochloa decumbens	Panicoideae	NC_030066
Urochloa brizantha	Panicoideae	NC_030067
