# Supplementary material for: Molecular dating of phylogenetic divergence between Urochloa species based on complete chloroplast genomes
Source: BMC Genomics. 2017 Jul 6;18:516. doi: 10.1186/s12864-017-3904-2 (PMC5499013; doi:10.1186/s12864-017-3904-2)
Supplement: Supplementary file 3 — Sequencing and de novo assembly metrics of chloroplast genomes of four Urochloa species. (PDF 13 kb) [file 12864_2017_3904_MOESM3_ESM.pdf]

Additional file 3 – Sequencing and *de novo* assembly metrics of chloroplast genomes of four *Urochloa* species

**Species**	**Reads (bp)**	**N50**	**Minimum size (bp)**	**Maximum size (bp)**	**Mean size (bp)**	**Contigs**	**Total (bp)**	**cpDNA assembly size (bp)**	**% recovery of assembled genome***
U. ruziziensis	20,211,010,488	1,704	200	57,461	754	382,380	288,171,438	136,733	98.5%
U. brizantha	8,643,705,720	505	200	86,745	464	380,899	176,721,916	134,165	96.5%
U. humidicola	8,476,910,040	485	200	80,478	448	476,044	213,428,620	138,213	99.45%
U. decumbens	9,018,811,776	491	200	38,791	455	408,401	185,680,658	129,056	92.89%

*Size of *de novo* assemblies when compared to assemblies using *P. virgatum* cpDNA as a reference
