# Supplementary material for: Molecular dating of phylogenetic divergence between Urochloa species based on complete chloroplast genomes
Source: BMC Genomics. 2017 Jul 6;18:516. doi: 10.1186/s12864-017-3904-2 (PMC5499013; doi:10.1186/s12864-017-3904-2)
Supplement: Supplementary file 4 — Annotation of chloroplast microsatellites in four Urochloa species. Genome locations are based on the plastid genome of U. brizantha. The table includes locations of cpSSRs relative to annotated genes, types of regions (exon, intron, intergenic), repeat motifs, physical position in bp, polymorphism information, and number of repeats in U. decumbens, U. ruziziensis, and U. humidicola. (PDF 44 kb) [file 12864_2017_3904_MOESM4_ESM.pdf]

Location	Region	Motif (*U. brizantha*)	SSR start1	SSR end	Polymorphism	*U. decumbens*	*U. ruziziensis*	*U. humidicola*
psbA	exon	(CTT/AAG)3	310	318	monomorphic			
psbA	exon	(AGA/TCT)3	403	411	monomorphic			
trnK-UUU-intron	intron	(T/A)10	3525	3534	polymorphic	(T/A)10	(T/A)9	(T/A)9
trnK-UUU-intron	intron	(T/A)12	3727	3738	polymorphic	(T/A)10	(T/A)10	(T/A)8
psbK-psbI	intergenic	(T/A)10	7145	7154	polymorphic	(T/A)10	(T/A)11	(T/A)7
psbC	exon	(GCT/AGC)3	10934	10942	polymorphic	(GCT/AGC)3	(GCT/AGC)2	(GCT/AGC)3
psbZ-trnG-UCC	intergenic	(TAAA/TTTA)3	11809	11820	polymorphic	(TAAA/TTTA)3	(TAAA/TTTA)1	(TAAA/TTTA)3
trnfM-CAU-trnT-GGU	intergenic	(A/T)10	12715	12724	polymorphic	(A/T)9	(A/T)8	(A/T)8
trnfM-CAU-trnT-GGU	intergenic	(ATA/TAT)3	14991	14999	monomorphic			
trnfM-CAU-trnT-GGU	intergenic	(ATA/TAT)3	15138	15146	polymorphic	(ATA/TAT)2	absent	absent
trnM-CAU-trnE-UUC	intergenic	(A/T)10	15337	15346	polymorphic	(A/T)9	(A/T)9	(A/T)8
trnD-GUC-psbM	intergenic	(ATAC/GTAT)3	17090	17101	monomorphic			
trnD-GUC-psbM	intergenic	(A/T)12	17432	17442	polymorphic	(A/T)6-T-(A/T)4	(A/T)9	(A/T)9
psbM-petN	intergenic	(A/T)11	17900	17910	polymorphic	(A/T)11	(A/T)10	(A/T)8
TrnC-GCA-rpoB	intergenic	(A/T)12	19521	19532	polymorphic	(A/T)9	(A/T)4-C-(A/T)7	(A/T)4-C-(A/T)8
TrnC-GCA-rpoB	intergenic	(CTA/TAG)3	19916	19924	monomorphic			
RpoB-rpoC1	intergenic	(AGA/TCT)3	20998	21006	monomorphic			
rpoC2	exon	(TAT/ATA)3	26120	26128	monomorphic			
rpoC2	exon	(AT/AT)5	26616	26625	monomorphic			
rpoC2	exon	(AGA/TCT)3	27802	27810	monomorphic			
rpoC2	exon	(AGA/TCT)3	28132	28140	undefined	(AGA/TCT)3	motif change (AGA)GGA(AGA)	motif change (AGA)GGA(AGA)
rpoC2	exon	(AGA/TCT)3	28144	28152	monomorphic			
rpoC2	exon	(AGA/TCT)3	28198	28206	monomorphic			
rpoC2	exon	(A/T)10	30336	30345	monomorphic			
rpoC2-rps2	intergenic	(ATT/AAT)3	30650	30658	monomorphic			
rpoC2-rps2	intergenic	(A/T)10	30732	30741	polymorphic	(A/T)21	(A/T)9	(A/T)8
atpI	exon	(GTT/AAC)3	32319	32327	monomorphic			
AtpI-atpH	intergenic	(A/T)12	33242	33253	polymorphic	(A/T)10	(A/T)10	(A/T)10
atpH	exon	(TGC/GCA)3	33285	33293	monomorphic			
psaA	exon	(TTG/CAA)3	41021	41029	monomorphic			
psaA	exon	(GCA/TGC)3	41314	41322	monomorphic			
psaA	exon	(TAG/CTA)3	41441	41449	undefined	(TAG/CTA)3	motif change (TAG)TTG(TAG)	motif change (TAG)TTG(TAG)
PsaA-ycf3	intergenic	(A/T)10	42643	42652	polymorphic	(A/T)11	(A/T)13	(A/T)13
ycf3	intron	(A/T)11	43783	43793	polymorphic	(A/T)11	(A/T)10	(A/T)8
ycf3	intron	(TCCT/AGGA)3	43916	43927	monomorphic			
ycf3	intron	(A/T)11	44990	45000	polymorphic	(A/T)11	(A/T)24	(A/T)17
ycf3	intron	(A/T)12	45002	45013	polymorphic	(A/T)11	(A/T)24	(A/T)17
TrnT-UGU-trnL-UAA	intergenic	(A/T)14	47400	47413	polymorphic	(A/T)12	(A/T)10	(A/T)4-A-(A/T)9
TrnT-UGU-trnL-UAA	intergenic	(A/T)10	47495	47504	polymorphic	(A/T)10	(A/T)9	(A/T)8
ndhK	exon	(ACC/GGT)3	50461	50469	monomorphic			
ndhK	exon	(A/T)15	50621	50635	polymorphic	(A/T)15	(A/T)15	(A/T)9
TrnM-CAU	Exon-intron	(GTAG/CATC)4	52885	52900	polymorphic	(GTAG/CATC)4	(GTAG/CATC)4	(GTAG/CATC)3
atpB	exon	(TCC/GGA)3	53661	53669	monomorphic			
AtpB-rbcL	intergenic	(AAC/GTT)3	55440	55448	monomorphic			
petA	exon	(TTC/GAA)3	61102	61110	monomorphic			
TrnP-UGG-psaJ	intergenic	(TA/TA)5	65236	65245	polymorphic	(TA/TA)5	(TA/TA)5	(TA/TA)6
TrnP-UGG-psaJ	intergenic	(A/T)10	65260	65269	polymorphic	(A/T)9	(A/T)12	(A/T)12
PsaJ-rpl33	intergenic	(TTC/GAA)4	65751	65762	undefined	(TTC/GAA)4	(TTC/GAA)4	motif change (TTC)T(TTC)
rpl33-rps18	intergenic	(TA/TA)5	66185	66194	polymorphic	(TA/TA)5	(TA/TA)5	(TA/TA)4
rpl33-rps18	intergenic	(A/T)11	66273	66283	polymorphic	(A/T)12	(A/T)13	(A/T)11
rps18	exon	(AAC/GTT)3	66560	66568	monomorphic			
rpl20-rps12	intergenic	(TAA/TTA)3	67449	67457	monomorphic			
rps12-exon-clpP	intergenic	(TCA/TGA)3	68249	68257	monomorphic			
ClpP-psbB	intergenic	(AGAA/TTCT)3	69058	69069	monomorphic			
PsbH-petB	intergenic	(A/T)10	72126	72135	polymorphic	(A/T)8	(A/T)8	(A/T)6-C-(A/T)3
PsbH-petB	intergenic	(A/T)11	72380	72390	polymorphic	(A/T)6	(A/T)6	(A/T)7
PetD-rpoA	intergenic	(A/T)11	74934	74944	polymorphic	(A/T)13	(A/T)15	(A/T)10
RpoA-rps11	intergenic	(TAT/ATA)3	76002	76010	monomorphic			
infA	exon	(A/T)11	77160	77170	polymorphic	(A/T)10	(A/T)10	(A/T)11
infA	exon	(A/T)10	77179	77188	monomorphic			
rp114	exon	(TAT/ATA)3	77962	77970	monomorphic			
rpl16	exon	(A/T)14	79248	79261	polymorphic	(A/T)7	(A/T)7	(A/T)7
rpl16	exon	(TTC/GAA)3	79343	79351	monomorphic			
rps3	exon	(TTC/GAA)3	79778	79786	monomorphic			
rpl22	exon	(TGC/GCA)3	80684	80692	monomorphic			
rpl22	exon	(TTC/GAA)3	80904	80912	monomorphic			
rpl12	exon	(TTC/GAA)3	81638	81646	monomorphic			
TrnI-CAU-trnL-CAA	intergenic	(AGG/CCT)3	85193	85201	monomorphic			
TrnI-CAU-trnL-CAA	intergenic	(AAG/CTT)3	85271	85279	monomorphic			
TrnI-CAU-trnL-CAA	intergenic	(A/T)10	86767	86776	polymorphic	(A/T)10	(A/T)10	(A/T)9
ndhB	exon	(AGA/TCT)3	88555	88563	monomorphic			
ndhB	exon	(AGA/TCT)3	90024	90032	monomorphic			
rps7-trnV-GAC	intergenic	(AAG/CTT)3	93126	93134	monomorphic			
rrn16	exon	(AAC/GTT)3	93914	93922	monomorphic			
TrnI-GAU	intron	(TCCT/AGGA)3	96110	96121	monomorphic			
Rrn4.5	exon	(AACG/CGTT)3	100751	100762	monomorphic			
NdhF-rpl32	intergenic	(AATG/CATT)3	106252	106263	polymorphic	(AATG/CATT)3	(AATG/CATT)4	(AATG/CATT)3
ccsA	exon	(A/T)10	108142	108151	polymorphic	(A/T)8	absent	(A/T)8
ndhD	exon	(AATA/TATT)3	108998	109009	monomorphic			
PsaC-ndhE	intergenic	(AAT/ATT)3	110810	110818	monomorphic			
PsaC-ndhE	intergenic	(TAT/ATA)3	111112	111120	monomorphic			
ndhA	exon	(TTC/GAA)3	113375	113383	monomorphic			
ndhH	exon	(ATCC/GGAT)3	115725	115736	monomorphic			
ndhH	exon	(TC/GA)5	115828	115837	monomorphic			
