# Supplementary material for: Molecular dating of phylogenetic divergence between Urochloa species based on complete chloroplast genomes
Source: BMC Genomics. 2017 Jul 6;18:516. doi: 10.1186/s12864-017-3904-2 (PMC5499013; doi:10.1186/s12864-017-3904-2)
Supplement: Supplementary file 5 — Annotation of SNPs located in genes in six pairwise comparisons between Urochloa species. The table includes genes, mutations, and SNP positions in bp. (PDF 46 kb) [file 12864_2017_3904_MOESM5_ESM.pdf]

U .brizantha x U .decumbens	
**Genes [mutation]**	**SNP position (*U. brizantha*)**
matK [A/G]	2969
rpoC1 [G/A]	25442
rpoC2 [A/G]	27773
rpoC2 [A/C]	27883
rpoC2 [A/G]	28097
rpoC2 [C/A]	28496
rpoC2 [A/C]	30347
ndhJ [A/C]	49530
rps18 [G/A]	66376
rps18 [A/C]	66752
rpoA [T/G]	75034
infA [T/G]	77160
ndhF [G/A]	105477
ccsA [A/G]	108124
ccsA [A/T]	108150
ccsA [A/G]	108151
ccsA [G/T]	108153
ndhG [G/A]	111854
ndhA [C/A]	114631

U .ruziziensis x U. decumbens	
**Genes [mutation]**	**SNP position (*U. ruziziensis*)**
psbA [G/C]	456
matK [C/T]	1789
matK [A/G]	1921
matK [C/T]	1922
matK [C/T]	1924
matK [C/A]	2430
matK [C/T]	2512
matK [G/T]	2729
matK [C/T]	2888
matK [T/G]	2947
matK [G/A]	2984
psbC [A/C]	10973
rpoB [T/C]	20329
rpoB [A/C]	20399
rpoB [G/A]	20400
rpoB [C/A]	21504
rpoB [A/G]	22089
rpoB [G/T]	23356
rpoC1 [C/T]	24440
rpoC1 [T/A]	24692
rpoC1 [T/C]	25153
rpoC1 [G/A]	25214
rpoC1 [A/C]	25319
rpoC2 [C/G]	25697
rpoC2 [G/A]	26273
rpoC2 [A/G]	26321
rpoC2 [A/G]	27196
rpoC2 [C/T]	27270

rpoC2 [C/T]	27416
rpoC2 [T/G]	27509
rpoC2 [A/G]	27545
rpoC2 [A/C]	27655
rpoC2 [A/G]	27663
rpoC2 [G/A]	27704
rpoC2 [C/A]	27806
rpoC2 [G/A]	28019
rpoC2 [G/A]	28242
rpoC2 [C/A]	28379
rpoC2 [A/G]	28704
rpoC2 [G/A]	28776
rpoC2 [T/C]	28913
rpoC2 [T/C]	29081
rpoC2 [A/T]	29291
rpoC2 [T/C]	30147
rps2 [T/G]	30855
psaA [A/G]	42294
ndhJ [A/C]	49293
ndhK [C/A]	49857
atpB [T/C]	53282
atpB [T/G]	54415
rbcL [G/A]	56354
petA [G/A]	60221
psaJ [A/G]	65146
psaJ [G/A]	65226
rps18 [T/C]	66142
rps18 [A/C]	66178
rpoA [T/G]	74811
rpoA [A/C]	75567
rps8 [A/G]	77382
rpl16 [T/C]	78174
rpl16 [C/T]	78206
rps3 [A/C]	80061
rps3 [A/G]	80078
rpl22 [T/C]	80319
rps7 [T/G]	90360
ndhF [T/A]	103762
ndhF [T/A]	103763
ndhF [A/G]	104021
ndhF [G/A]	104401
ndhF [C/T]	104615
ndhF [C/A]	105018
ndhF [A/G]	105031
ndhF [C/T]	105152
ndhF [G/A]	105283
ndhF [C/T]	105556
rpl32 [T/C]	106575
ccsA [G/T]	107695
ccsA [T/G]	107932
ccsA [T/A]	107933
ccsA [T/C]	107937

ndhD [C/A]	109709
ndhD [C/T]	110063
ndhE [G/C]	111212
ndhG [G/A]	111636
ndhA [G/T]	112927
ndhA [G/T]	113032
ndhA [C/A]	114439
ndhA [T/C]	114745

U. ruziziensis x U. brizantha	
**Genes [mutation]**	**SNP position (*U. ruziziensis*)**
psbA [G/C]	456
matK [C/T]	1789
matK [A/G]	1921
matK [C/T]	1922
matK [C/T]	1924
matK [C/A]	2430
matK [C/T]	2512
matK [G/T]	2729
matK [C/T]	2888
matK [T/G]	2947
matK [G/A]	2983
matK [G/A]	2984
psbC [A/C]	10973
rpoB [T/C]	20329
rpoB [A/C]	20399
rpoB [G/A]	20400
rpoB [C/A]	21504
rpoB [A/G]	22089
rpoB [G/T]	23356
rpoC1 [C/T]	24440
rpoC1 [T/A]	24692
rpoC1 [T/C]	25153
rpoC1 [A/C]	25319
rpoC2 [C/G]	25697
rpoC2 [G/A]	26273
rpoC2 [A/G]	26321
rpoC2 [A/G]	27196
rpoC2 [C/T]	27270
rpoC2 [C/T]	27416
rpoC2 [T/G]	27509
rpoC2 [A/G]	27663
rpoC2 [G/A]	27704
rpoC2 [C/A]	27806
rpoC2 [G/A]	27869
rpoC2 [G/A]	28019
rpoC2 [G/A]	28242
rpoC2 [A/C]	28268
rpoC2 [C/A]	28379
rpoC2 [A/G]	28704
rpoC2 [G/A]	28776

rpoC2 [T/C]	28913
rpoC2 [T/C]	29081
rpoC2 [A/T]	29291
rpoC2 [C/A]	30119
rpoC2 [T/C]	30147
rps2 [T/G]	30855
psaA [A/G]	42294
ndhK [C/A]	49857
atpB [T/C]	53282
atpB [T/G]	54415
rbcL [G/A]	56354
rbcL [T/A]	56869
rbcL [A/G]	56870
rbcL [A/T]	56872
rbcL [A/C]	56887
petA [G/A]	60221
psaJ [A/G]	65146
psaJ [G/A]	65226
rps18 [T/C]	66142
rps18 [A/G]	66164
rps18 [A/C]	66178
rps18 [C/A]	66539
rpoA [A/C]	75567
infA [G/T]	76939
rps8 [A/G]	77382
rpl16 [T/C]	78174
rpl16 [C/T]	78206
rps3 [A/C]	80061
rps3 [A/G]	80078
rpl22 [T/C]	80319
rps7 [T/G]	90360
ndhF [T/A]	103762
ndhF [T/A]	103763
ndhF [A/G]	104021
ndhF [G/A]	104401
ndhF [C/T]	104615
ndhF [C/A]	105018
ndhF [A/G]	105031
ndhF [C/T]	105152
ndhF [C/T]	105556
rpl3 [T/C]	106575
ccsA [G/T]	107695
ccsA [T/A]	107932
ccsA [T/A]	107933
ccsA [T/C]	107937
ndhD [C/A]	109709
ndhD [C/T]	110063
ndhE [G/C]	111212
ndhA [G/T]	112927
ndhA [G/T]	113032
ndhA [T/C]	114745

U. humidicola x U. ruziziensis	
**Genes [mutation]**	**SNP position (*U. humidicola*)**
psbA [C/G]	459
matK [T/C]	1689
matK [T/C]	1797
matK [C/G]	1918
matK [G/A]	1929
matK [T/C]	1930
matK [C/T]	2151
matK [A/C]	2321
matK [G/A]	2326
matK [A/C]	2437
matK [A/C]	2438
matK [T/G]	2737
matK [A/G]	2992
matK [A/C]	3000
matK [G/T]	3075
matK [G/A]	3145
psbC [T/C]	10283
psbC [C/A]	11093
rpoB [A/C]	20460
rpoB [C/G]	20592
rpoB [C/T]	20619
rpoB [A/G]	20690
rpoB [A/G]	20852
rpoB [T/C]	21294
rpoB [T/G]	21775
rpoB [A/C]	21794
rpoB [A/G]	22280
rpoB [G/A]	22379
rpoB [A/G]	22988
rpoC1 [G/A]	23793
rpoC1 [G/T]	24982
rpoC1 [A/G]	25002
rpoC1 [C/A]	25386
rpoC1 [A/G]	25392
rpoC1 [C/T]	25443
rpoC1 [A/T]	25445
rpoC1 [G/A]	25453
rpoC1 [C/T]	25540
rpoC1 [A/G]	25546
rpoC1 [C/A]	25609
rpoC2 [A/G]	26563
rpoC2 [A/G]	26612
rpoC2 [A/G]	26764
rpoC2 [G/A]	27486
rpoC2 [C/A]	27498
rpoC2 [T/G]	27601
rpoC2 [T/C]	27709
rpoC2 [G/T]	27802
rpoC2 [G/T]	28092

rpoC2 [G/T]	28097
rpoC2 [G/C]	28102
rpoC2 [A/G]	28124
rpoC2 [A/G]	28514
rpoC2 [A/G]	28538
rpoC2 [A/C]	28675
rpoC2 [A/C]	28767
rpoC2 [G/A]	28799
rpoC2 [A/C]	29287
rpoC2 [C/T]	29383
rpoC2 [C/G]	29410
rpoC2 [A/G]	29413
rpoC2 [G/A]	29501
rpoC2 [A/G]	29661
rpoC2 [C/A]	29744
rpoC2 [A/G]	30505
atpF [T/C]	34092
psaA [G/A]	42607
ndhJ [T/G]	49624
ndhK [T/C]	50079
ndhK [G/C]	50135
ndhK [A/C]	50177
atpB [C/T]	53589
atpB [T/C]	54986
ycf4 [T/A]	58762
ycf4 [A/G]	58847
ycf4 [G/A]	58893
cemA [C/G]	59402
cemA [C/G]	59622
cemA [A/G]	59649
cemA [G/A]	59675
petA [A/C]	60728
psaJ [G/A]	65443
psaJ [A/G]	65523
rpl33 [T/C]	65996
rps18 [C/T]	66429
rps18 [A/G]	66430
rps18 [A/C]	66444
rps18 [A/C]	66723
rps18 [T/C]	66774
rps18 [A/C]	66830
rpl20 [T/C]	67188
psbN [A/G]	71526
rpoA [T/C]	75276
rpoA [T/G]	75333
rpoA [T/C]	75334
infA [T/G]	77209
rps8 [G/A]	77554
rps8 [G/A]	77653
rps8 [T/G]	77701
rpl16 [C/T]	78444
rps3 [G/A]	80299

rpl2 [T/C]	81657
ndhF [T/C]	103968
ndhF [A/C]	103979
ndhF [T/G]	104051
ndhF [G/T]	104153
ndhF [G/T]	104211
ndhF [G/A]	104236
ndhF [A/C]	104364
ndhF [C/T]	104451
ndhF [A/G]	104616
ndhF [C/A]	104789
ndhF [T/C]	104830
ndhF [A/C]	105028
rpl32 [C/T]	106793
rpl32 [C/T]	106856
ccsA [A/G]	107739
ccsA [C/T]	107772
ccsA [C/A]	107951
ccsA [C/A]	107992
ccsA [G/T]	108140
ccsA [A/T]	108141
ccsA [A/T]	108151
ccsA [C/T]	108180
ccsA [C/T]	108246
ccsA [C/A]	108255
ndhD [A/C]	109928
ndhE [C/G]	111449
ndhG [A/C]	112158
ndhG [A/C]	112159
ndhG [G/A]	112171
ndhI [A/C]	112410
ndhA [T/G]	113162
ndhA [A/C]	113499
ndhA [A/G]	114829
ndhA [C/T]	114958
ndhH [G/A]	116252

U. humidicola x U. brizantha	
**Genes [mutation]**	**SNP position (*U. humidicola*)**
matK [T/C]	1689
matK [C/G]	1918
matK [C/T]	1932
matK [C/T]	2151
matK [A/T]	2321
matK [G/A]	2326
matK [A/C]	2437
matK [C/T]	2520
matK [C/T]	2896
matK [T/G]	2955
matK [G/A]	2991
matK [A/C]	3000

matK [G/T]	3075
matK [G/A]	3145
psbC [T/C]	10283
rpoB [A/C]	20460
rpoB [C/G]	20592
rpoB [A/C]	20689
rpoB [A/G]	20852
rpoB [T/C]	21294
rpoB [T/G]	21775
rpoB [A/G]	22280
rpoB [A/G]	22988
rpoB [G/T]	23646
rpoC1 [G/A]	23793
rpoC1 [C/T]	24730
rpoC1 [G/A]	24982
rpoC1 [A/G]	25002
rpoC1 [C/A]	25386
rpoC1 [A/G]	25392
rpoC1 [A/T]	25445
rpoC1 [G/A]	25453
rpoC1 [C/T]	25540
rpoC1 [A/G]	25546
rpoC2 [C/G]	25987
rpoC2 [A/G]	26611
rpoC2 [A/G]	26612
rpoC2 [A/G]	26764
rpoC2 [C/A]	27498
rpoC2 [C/T]	27560
rpoC2 [T/G]	27601
rpoC2 [A/G]	27959
rpoC2 [G/A]	28000
rpoC2 [G/T]	28092
rpoC2 [G/T]	28097
rpoC2 [G/A]	28102
rpoC2 [A/G]	28124
rpoC2 [G/A]	28165
rpoC2 [G/A]	28315
rpoC2 [A/G]	28514
rpoC2 [A/C]	28564
rpoC2 [A/C]	28767
rpoC2 [G/A]	28799
rpoC2 [A/G]	29000
rpoC2 [G/A]	29072
rpoC2 [T/C]	29215
rpoC2 [A/C]	29287
rpoC2 [C/G]	29410
rpoC2 [A/G]	29413
rpoC2 [G/A]	29501
rpoC2 [A/T]	29593
rpoC2 [A/G]	29661
rpoC2 [C/A]	29744
rpoC2 [C/A]	30421

rpoC2 [T/C]	30449
rpoC2 [A/G]	30505
rps2 [T/G]	31158
atpF [T/C]	34092
ndhJ [T/G]	49624
ndhK [T/C]	50079
ndhK [G/C]	50135
atpB [T/G]	54722
atpB [T/C]	54986
rbcL [G/A]	56665
ycf4 [T/A]	58762
ycf4 [A/G]	58847
ycf4 [G/A]	58893
cemA [C/G]	59402
cemA [C/G]	59622
cemA [A/G]	59649
cemA [G/A]	59675
petA [G/A]	60524
petA [A/C]	60728
rpl33 [T/C]	65996
rps18 [A/G]	66430
rps18 [A/C]	66444
rps18 [A/G]	66451
rps18 [A/C]	66465
rps18 [A/C]	66723
rps18 [T/C]	66774
rps18 [C/A]	66826
rps18 [A/C]	66830
rpl20 [T/C]	67188
psbN [A/G]	71526
rpoA [T/C]	75276
rpoA [T/G]	75333
rpoA [T/C]	75334
rpoA [A/C]	75841
rps8 [G/A]	77554
rps8 [T/G]	77701
rpl16 [C/T]	78476
rps3 [A/C]	80282
rpl22 [T/C]	80539
rpl2 [T/C]	81657
rps7 [T/G]	90576
ndhF [T/C]	103968
ndhF [T/A]	103977
ndhF [T/A]	103978
ndhF [A/C]	103979
ndhF [T/G]	104051
ndhF [G/T]	104153
ndhF [G/T]	104211
ndhF [A/C]	104364
ndhF [C/T]	104451
ndhF [C/A]	104789
ndhF [A/C]	105028

ndhF [C/A]	105233
ndhF [A/G]	105246
ndhF [C/T]	105367
ndhF [C/T]	105771
rpl32 [C/T]	106856
ccsA [A/G]	107739
ccsA [C/T]	107772
ccsA [G/T]	107908
ccsA [C/A]	107951
ccsA [C/A]	107992
ccsA [G/A]	108140
ccsA [A/T]	108151
ccsA [T/A]	108166
ccsA [G/A]	108167
ccsA [C/T]	108180
ccsA [A/G]	108234
ccsA [C/T]	108246
ccsA [C/A]	108255
ndhD [C/T]	110282
ndhG [A/C]	112158
ndhG [A/C]	112159
ndhG [G/A]	112171
ndhI [A/C]	112410
ndhA [G/T]	113267
ndhA [A/C]	113499
ndhA [A/G]	114829
ndhH [G/A]	116252

U. humidicola x U. decumbens	
**Genes [mutation]**	**SNP position (*U. humidicola*)**
matK [T/C]	1689
matK [C/G]	1918
matK [C/T]	1932
matK [C/T]	2151
matK [A/T]	2321
matK [G/A]	2326
matK [A/C]	2437
matK [C/T]	2520
matK [C/T]	2896
matK [T/G]	2955
matK [A/C]	3000
matK [G/T]	3075
matK [G/A]	3145
psbC [T/C]	10283
rpoB [A/C]	20460
rpoB [C/G]	20592
rpoB [A/C]	20689
rpoB [A/G]	20852
rpoB [T/C]	21294
rpoB [T/G]	21775
rpoB [A/G]	22280

rpoB [A/G]	22988
rpoB [G/T]	23646
rpoC1 [G/A]	23793
rpoC1 [C/T]	24730
rpoC1 [G/A]	24982
rpoC1 [A/G]	25002
rpoC1 [C/A]	25386
rpoC1 [A/G]	25392
rpoC1 [A/T]	25445
rpoC1 [G/A]	25453
rpoC1 [G/A]	25504
rpoC1 [C/T]	25540
rpoC1 [A/G]	25546
rpoC2 [C/G]	25987
rpoC2 [A/G]	26611
rpoC2 [A/G]	26612
rpoC2 [A/G]	26764
rpoC2 [C/A]	27498
rpoC2 [C/T]	27560
rpoC2 [T/G]	27601
rpoC2 [A/G]	27838
rpoC2 [A/C]	27951
rpoC2 [A/G]	27959
rpoC2 [G/A]	28000
rpoC2 [G/T]	28092
rpoC2 [G/T]	28097
rpoC2 [G/A]	28102
rpoC2 [A/G]	28124
rpoC2 [G/A]	28315
rpoC2 [A/G]	28514
rpoC2 [A/C]	28767
rpoC2 [G/A]	28799
rpoC2 [A/G]	29000
rpoC2 [G/A]	29072
rpoC2 [T/C]	29215
rpoC2 [A/C]	29287
rpoC2 [C/G]	29410
rpoC2 [A/G]	29413
rpoC2 [G/A]	29501
rpoC2 [A/T]	29593
rpoC2 [A/G]	29661
rpoC2 [C/A]	29744
rpoC2 [T/C]	30449
rpoC2 [A/G]	30505
rps2 [T/G]	31158
atpF [T/C]	34092
ndhJ [A/C]	49617
ndhJ [T/G]	49624
ndhK [T/C]	50079
ndhK [G/C]	50135
atpB [T/G]	54722
atpB [T/C]	54986

rbcL [G/A]	56665
ycf4 [T/A]	58762
ycf4 [A/G]	58847
ycf4 [G/A]	58893
cemA [C/G]	59402
cemA [C/G]	59622
cemA [A/G]	59649
cemA [G/A]	59675
petA [G/A]	60524
petA [A/C]	60728
rpl33 [T/C]	65996
rps18 [A/G]	66430
rps18 [A/C]	66444
rps18 [A/C]	66465
rps18 [A/C]	66723
rps18 [T/C]	66774
rps18 [A/C]	66830
rpl20 [T/C]	67188
psbN [A/G]	71526
rpoA [T/G]	75085
rpoA [T/C]	75276
rpoA [T/G]	75333
rpoA [T/C]	75334
rpoA [A/C]	75841
infA [T/G]	77209
rps8 [G/A]	77554
rps8 [T/G]	77701
rpl16 [C/T]	78476
rps3 [A/C]	80282
rpl22 [T/C]	80539
rpl2 [T/C]	81657
rps7 [T/G]	90576
ndhF [T/C]	103968
ndhF [T/A]	103977
ndhF [T/A]	103978
ndhF [A/C]	103979
ndhF [T/G]	104051
ndhF [G/T]	104153
ndhF [G/T]	104211
ndhF [A/C]	104364
ndhF [C/T]	104451
ndhF [C/A]	104789
ndhF [A/C]	105028
ndhF [C/A]	105233
ndhF [A/G]	105246
ndhF [C/T]	105367
ndhF [G/A]	105498
ndhF [C/T]	105771
rpl32 [C/T]	106856
ccsA [A/G]	107739
ccsA [C/T]	107772
ccsA [G/T]	107908

ccsA [C/A]	107951
ccsA [C/A]	107992
ccsA [A/T]	108151
ccsA [C/T]	108180
ccsA [A/G]	108234
ccsA [C/T]	108246
ccsA [C/A]	108255
ndhD [C/T]	110282
ndhG [G/A]	111873
ndhG [A/C]	112158
ndhG [A/C]	112159
ndhG [G/A]	112171
ndhI [A/C]	112410
ndhA [G/T]	113267
ndhA [A/C]	113499
ndhA [C/A]	114652
ndhA [A/G]	114829
ndhH [G/A]	116252
