# Supplementary material for: Molecular dating of phylogenetic divergence between Urochloa species based on complete chloroplast genomes
Source: BMC Genomics. 2017 Jul 6;18:516. doi: 10.1186/s12864-017-3904-2 (PMC5499013; doi:10.1186/s12864-017-3904-2)
Supplement: Supplementary file 6 — Total number of InDels, their effects, and genome locations in six pairwise comparisons between Urochloa species. InDels located in genes are listed with their respective variations (insertion/deletion) and genome positions. (PDF 23 kb) [file 12864_2017_3904_MOESM6_ESM.pdf]

**Pairwise comparison**	***U. brizantha x U. decumbens***
Total number of InDels	91
Number of Effects	92
Region	
Intergenic	80
Intragenic	12
Intron	10
Exon	2
Type	
Frameshift variant	2
Stop lost	1
Genes [variation]	InDel position (U. brizantha)
rbcL [insertion – AAGGTCTAAATAAAATAAA]	57094
rps18 [insertion – T]	66381

**Pairwise comparison**	***U. ruziziensis x U. decumbens***
Total number of InDels	187
Number of Effects	188
Region	
Intergenic	164
Intragenic	19
Intron	14
Exon	5
Type	
Frameshift variant	2
Stop lost	1
Stop gained	1
Inframe insertion	1
Inframe deletion	1
Disruptive inframe insertion	1
Genes [variation]	InDel position (U. ruziziensis)
rbcL [insertion – AGAAAATAAAAAAGAAGAGAA]	56870
rbcL [insertion – AG]	56893
ccsA [insertion – AGAAT]	107919
ccsA [deletion – ATGTTT]	107956
ccsA [deletion – AAG]	107957

**Pairwise comparison**	***U. ruziziensis x U. brizantha***
Total number of InDels	192
Number of Effects	192
Region	
Intergenic	161
Intragenic	26
Intron	19
Exon	7
Type	
Frameshift variant	4
Stop gained	2
Inframe insertion	1
Disruptive inframe insertion	2
Genes [variation]	InDel position (U. ruziziensis)
rbcL [deletion – TC]	56871
rbcL [deletion – T]	56882
rps18 [deletion – T]	66168
ccsA [insertion – TAGAA]	107923
ccsA [deletion – AAATGT]	107956
ccsA [deletion – AAG]	107957

**Pairwise comparison**	***U. humidicola x U. ruziziensis***
Total number of InDels	268
Number of Effects	268
Region	
Intergenic	228
Intragenic	30
Intron	20
Exon	10
Type	
Frameshift variant	6
Stop lost	1
Inframe insertion	1
Disruptive inframe insertion	1
Inframe deletion	2
Genes [variation]	InDel position (U. humidicola)
rpoC2 [insertion – CAA]	27579
rpoC2 [insertion – CC]	27905
rpoC2 [insertion – G]	27911
rpoC2 [insertion – AAGA]	29189
rpoC2 [insertion – AC]	29195
ndhK [deletion – TTTTTT]	50715
rbcL [insertion – T]	57179

ccsA [deletion – TAGAA]	108135
ccsA [insertion – AAG]	108172

**Pairwise comparison**	***U. humidicola x U. brizantha***
Total number of InDels	259
Number of Effects	259
Region	
Intergenic	223
Intragenic	27
Intron	19
Exon	8
Type	
Frameshift variant	5
Stop lost	1
Inframe insertion	1
Inframe deletion	2
Genes [variation]	InDel position (U. humidicola)
rpoC2 [insertion – CAA]	27582
rpoC2 [insertion – CC]	27905
rpoC2 [insertion – G]	27911
rpoC2 [insertion – AAGA]	29189
rpoC2 [insertion – AC]	29195
ndhK [deletion – TTTTTT]	50715
rbcL [insertion – ATA]	57178
rps18 [deletion – T]	66455

**Pairwise comparison**	***U. humidicola x U. decumbens***
Total number of InDels	258
Number of Effects	259
Region	
Intergenic	223
Intragenic	26
Intron	19
Exon	7
Type	
Frameshift variant	4
Stop lost	1
Inframe insertion	1
Inframe deletion	2
Genes [variation]	InDel position (U. humidicola)
rpoC2 [insertion – CAA]	27582
rpoC2 [insertion – CC]	27905

rpoC2 [insertion – G]	27911
rpoC2 [insertion – AAGA]	29189
rpoC2 [insertion – AC]	29195
ndhK [deletion – TTTTTT]	50715
rbcL [insertion – ATAAAGGAGGTCCAAATAA]	57178
